# A Case of Hysterectomy for Fibroid Tumour of Uterus, with Intra-Abdominal Treatment of Pedicle
3Paper read before the Bristol Medico-Chirurgical Society, January 11th, 1899.


**Published:** 1899-03

**Authors:** W. H. C. Newnham

**Affiliations:** Physician-Accoucheur, Bristol General Hospital.


### A CASE OF HYSTERECTOMY FOR FIBROID TUMOUR OF UTERUS, WITH INTRA-ABDOMINAL TREATMENT OF PEDICLE.[3]

BY

W. H. C. Newnham, M.A., M.B. Cantab.,

*Physician-Accoucheur, Bristol General Hospital.*

---

I have put together the notes of this case thinking it would be of interest to the Society, inasmuch as it is the first operation of this description that has been done in Bristol.

It is a far more satisfactory operation than the old one of treating the pedicle with some kind of mechanical clamp outside the abdominal wall, for the following reasons:—

(1) It is absolutely aseptic, while in the old operation there was always danger of suppuration of the pedicle.

(2) No mechanical means are required for arresting hemorrhage, as all vessels are tied at the time of division.

(3) There is no drag on the pedicle and practically no risk of peritonitis.

[3] Paper read before the Bristol Medico-Chirurgical Society, January 11th, 1899.

(4) Bland Sutton asserts (and certainly has proved it in his own operations) that this method possesses no more risk than an ordinary ovariotomy. The latest statistics shew that the mortality is less than it was with the old fashioned operation, which the late Mr. Greig Smith clung to even to the end of his life.

The patient, M. H., aged 35, was sent to me by Dr. Freeman on November 1st, 1898. She had had two children, the last ten years ago. Menstruation commenced at the age of 13, always lasted five to six days, and was always excessive.

About a year before admission patient had noticed some swelling of the abdomen; at the same time she had great pain in this region, which varied in intensity. Four months ago she noticed the swelling had increased considerably in size and was causing still more pain: her periods also now became more excessive.

On examination there was a large, soft, semi-fluctuating tumour to be felt in the median line of the abdomen, rising out of the pelvis. It was extremely painful if handled at all; the sound passed three inches, and the tumour was apparently in front of the uterus itself. By some of my colleagues it was thought it might be the bladder or some diverticulum of that organ.

On November 10th, the patient being placed under ether, I made a long incision into the abdominal cavity, and at once brought out a large fibroid tumour (produced) growing from the fundus of the uterus. A pedicle needle threaded with stout silk was passed through the broad ligament, and the ovarian arteries and veins were ligatured on each side and cut away from the uterus. The ovaries were left untouched, this being considered a very important part of the operation. It is not at all necessary to remove the ovaries, and it is considered much better for the patient to retain them if possible.

The pedicle needle was now pushed through the broad ligament on each side below the uterine arteries, and these were ligatured after some slight difficulty. This appears to be the most difficult step of the operation.

A semi-circular incision was now made with the convexity upwards on the anterior aspect of the uterus, below the point where the tumour joined the body of the uterus: a similar flap was made posteriorly in the same manner, and then the tumour and upper part of the uterus were cut away, carrying the knife down deeply below the flaps and taking away as much as possible. A few bleeding points in the flaps were tied, and then they were brought into exact apposition by means of silk sutures sewn deeply through the flaps. Then, there being no hemorrhage, the pedicle was dropped into the pelvis and the abdominal wall sutured in the ordinary manner.

The patient was put to bed, and from that time had not a bad symptom. She was able to get up on November 28th, or eighteen days after the operation.

Now, is not this a contrast to the old hysterectomy, with a sloughing pedicle hanging out from the abdominal wall with foul pus discharging from the stump, the chance of peritonitis from dragging on the pedicle, the risk of hemorrhage which may easily be fatal from slipping or loosening of the clamp, the danger of some drop or so of foul pus getting into the peritoneal cavity, the separation and final healing of the stump not taking place for many weary weeks? Whereas this patient runs no risk, but is well and walking about on the eighteenth day after the operation.

---

In the discussion on Dr. Newnham's case, Dr. Aust Lawrence stated that this mode of operation would in the future in a good many cases be preferred to the old plan of fixing the stump outside the abdomen, and he looked forward to the mortality being very much reduced in this class of operation. Dr. Aust Lawrence, however, spoke against any operation being done in a large number of cases of myoma uteri, as the symptoms did not warrant interference in at least 80 per cent. of the cases.—Mr. Roger Williams suggested that the operation done in this case might more appropriately be called " Abdominal myomectomy," since nothing but the tumour was removed. He was glad to see that the importance of preserving as much as possible of the reproductive organs was now beginning to be recognised. For the treatment of a non-malignant disease like myoma of the uterus, he thought myomectomy was a much superior operation to hysterectomy, which should be reserved for a few exceptional cases not otherwise operable. After myomectomy, women might bear healthy children; and with Zweifel's *technique*, the mortality of myomectomy was only about 5 per cent.—Dr. Walter Swayne, recalled to the recollection of those present a paper by Stuart Nairne, of Glasgow *(Prov. Med. Journ.*, 1889*)*, in which he not only dealt in this manner with the stump after hysterectomy, but related a long series of cases of myomectomy with most successful results. The retaining of the ovaries, if healthy, apart from the quicker convalescence, was a great advantage of the more modern method of operating. In considering the relations between the success of the intraperitoneal and extraperitoneal methods of treatment, it should be remembered that many of the former cases were operated on for small tumours, and the results were likely to be superior to those in cases where large tumours with dense adhesions existed; apart from this, however, the more modern method was undoubtedly superior to the old, and no doubt would become generally adopted in the future.—Mr. Carwardine spoke in favour of hysterectomy by retroperitoneal treatment of the stump when possible.—Mr. Morton thought that though the treatment of the pedicle by constriction with a wire in the extraperitoneal method seemed a barbarous and unsurgical proceeding, yet it was well to remember that it had a smaller mortality than the intraperitoneal method, and that was the main consideration. We could not, of course, judge of the respective merits of the two methods unless we had a series of cases treated by each; one case was of no value for such a purpose. He thought the best plan was to begin the operation with everything prepared for either method, and then to be guided by the condition of the growth. He thought, from an examination of the specimen, that Dr. Newnham's case was one particularly suited for the intraperitoneal method.—In reply, Dr. Newnham said: It is evident that Mr. Morton has not seen the latest statistics on the subject of mortality, for in the hands of

Bland Sutton and some German surgeons the mortality is reduced to the same level as the rate for ordinary ovariotomy. It is manifestly very easy to open the abdomen and remove a fibroid with an ordinary thin pedicle; but the difficulty arises when it is necessary to remove the tumour and the greater part of the uterus itself, the one not being capable of being distinguished from the other.

---

### SYMPHYSIOTOMY.

BY

WALTER C. SWAYNE, M.D. Lond.,

